# Intimate partner violence experienced by women living with—and without—disability in the European Union. A quantitative intersectional analysis

**DOI:** 10.3389/fsoc.2022.948811

**Published:** 2022-08-22

**Authors:** Pilar Rodriguez Martinez

**Affiliations:** Departamento de Historia, Geografia y Humanidades, Universidad de Almería, Almería, Spain

**Keywords:** intersectionality, intimate partner or ex-partner physical violence, women living with disabilities, European Union, quantitative analysis

## Abstract

This article aims to explore the specific combination and interactions of inequalities associated with experiencing Intimate Partner Violence that affects European women living with—or without—a disability. The analysis is based on the survey carried out by the European Union Agency for Fundamental Rights (FRA) between 2010 and 2012. In that survey, 42,002 women were interviewed, with a representative sample of women from each of the 28 countries of the European Union. We try to elucidate through a multiple logistic regression analysis if the experience of IPV is something that can simply be added to**—**or overlap with**—**social categories (feeling of household income, educational level, and marital status), the aggressor (partner abusing alcohol, partner violent against others), the relationship (duration, equal say in economic decisions), and the previous experience of IPV. The result of the analysis shows that living with a disability intersects with low income, which comes hand in hand with experiencing more violence. Other interactions like living with a disability when the woman is under 15 years and living with a disability and the partner abusing alcohol are also relevant. In terms of social policies, our result should induce investment in IPV prevention policies among poor women who live with a disability, who have a history of physical violence, and whose partners abuse alcohol.

## Introduction

Intimate Partner Violence (IPV) refers to behavior by an intimate partner or ex-partner that causes physical, sexual, or psychological harm, including physical aggression, sexual coercion, psychological abuse, and controlling behaviors. Worldwide, almost one-third (27%) of women aged 15–49 years report that they have suffered IPV (World Health Organization, 2021). Disability is a part of the human condition; it results from the interaction between individuals with a health condition, such as cerebral palsy, Down syndrome, and depression, with personal and environmental factors including negative attitudes, inaccessible transportation and public buildings, and limited social support. According to the World Report on Disability (World Health Organization, 2011), over 15% of the world's population lives with some type of disability, and among them, between 2 and 4% have significant difficulties functioning in daily life. It is estimated that 54% of disabled women experience violence by a partner or ex-partner in their lifetime (Campbell et al., 2022).

Living with a disability is associated with increased vulnerability (Hughes et al., 2012; Meyer et al., 2022), so increasing knowledge of the psychosocial and socio-cognitive factors that may be related to gender, culture, and biology is critical to understanding women's experiences (Patrao et al., 2015). Disability is highest among the lowest-income countries; it has a greater effect on the poorest quintile of the population: those who do not have a paid job, those with little educational training, the elderly, and women. It is linked to poorer academic results, lower levels of economic participation, greater dependency, and limited participation (World Health Organization, 2011). In short, living with a disability is associated with a systematic lack of economic and social resources in contemporary societies.

Living with a disability is fluid and comes with intersectional social division (Sommo and Chaskes, 2013). It has to do with a narrative, in which we must start from the subjective meaning of those who experience this social division (Green and Loseke, 2020), to understand this particular physical and social vulnerability (Ballan and Freyer, 2017). But not only that, it has to do with societal myths of categorizing these women as single and asexual (Barnett et al., 2005) which probably explains the lack of attention on IPV against women living with a disability (Brownridge, 2006, p. 806). It also has to do with a medical model which emphasizes the impairment of individual function, and its effects on individuals, with the accompanying focus on dependency (Oliver, 1990; Thiara et al., 2011).

Studies on disability highlight that, like sex-gender, living with a disability has to do with the person's relationship with social divisions in the whole social environment (Garland-Thomson, 2005, 2013; Shakespeare, 2014; Muster, 2020). This can be understood as oppression “to the extent that those without disability create a world that limits access to the channels of social, economic, and political power through the assignment of devalued social roles and discriminatory practices” (Sommo and Chaskes, 2013, p. 48).

The purpose of the present study is to examine the social category “living with/without a disability” in terms of its impact on the risk of IPV, with an emphasis on the intersections between this category and the rest of the social divisions. We will focus the present study specifically on the inter-categorical level of the intersectional analysis. In that level of analysis, we will document the relationships of inequality across social categories and present the result of logistic regression to explore how living with a disability intersects with these social categories in the situation of women who have experienced IPV in the European Union. Let us start summarizing the kinds of IPV that women living with a disability experience according to the literature. We will outline a general framework to situate our hypotheses and findings.

## Women living with a disability and intimate partner violence

Research highlights that women living with a disability can experience “compound oppression” (Nixon, 2009). They experience violence in a different and specific way, but above all, they experience IPV more frequently than women living without a disability (Brownridge, 2006; Copel, 2006; Veloo, 2006; Humphreys, 2007; Smith, 2007; Barrett et al., 2009; Hughes et al., 2011, 2012; Austin et al., 2014; Ballan and Freyer, 2017; Cotter, 2018; Naples et al., 2019; Muster, 2020). First of all, women living with a disability experience specific forms of violence related to IPV. As Austin, Lews, and Washington note, in a review of the literature on disabilities and interpersonal violence (2014), researchers have found cases of violence that only affect women with disabilities. It suggests that there is “disability-specific abuse” related to IPV. These authors highlight examples such as overmedication or under medication, neglect in the provision of essential personal care, and destruction or manipulation of assistive devices (Lund, 2011; Austin et al., 2014, p. 44) or macroaggression as brief, everyday slights, snubs, or insults (Barber et al., 2019).

In addition, some authors highlight that disability could alter the experience of IPV. An example of qualitative differences would be studies that have tried to compare and contrast the Walker Cycle of intimate partner violence (Copel, 2006) or the variations in the timing of first sexual experiences (Khan et al., 2019). As Copel points out, in the case of women living with a disability, the usual cycle of violence does not have a “honeymoon” phase after episodes of ill-treatment, and thus it could be argued that the experience of intimate partner violence of women living with a disability is different to the experiences described in traditional studies dealing with women living without a disability. Furthermore, scholars highlight the typical severity of physical abuse that women living with a disability suffered (Brownridge, 2006; Thiara et al., 2011; Coston, 2019; McGowan and Elliott, 2019), the hyperviolent and hypersexual nature of disability hate crime, and physical and sexual aggression (Sherry, 2010). For women living with a disability, the intensity of IPV may be greater.

And, finally, women living with a disability suffer IPV more frequently than women living without disabilities. For example, in the USA, the percentage of women living with a disability that suffer violent crimes triplicates the percentage of women without a disability, according to the 2013 National Crime Victimization Survey (Harrell, 2015), and according to the 2010 National Intimate Partner and Sexual Violence Survey, the percentage of women living with a disability who have suffered intimate partner violence was 23.8%. The association between disability status and Intimate Partner Victimization for physical partner violence reached an Adjusted Odds Ratio of 2.2 controlling with variables for age, family income, race or ethnicity, and education (Breiding and Armour, 2015). Smith, working with the data of the 2005 Behavioral Risk Factor Surveillance System found that in the USA, the variables increasing the likelihood of abuse include the unemployed, uncoupled, and young age (Smith, 2007). Examining the impact of disability status in two waves of the National Epidemiologic Survey, Hahn et al. (2014) found that women with physical and mental health impairments experience higher levels of IPV victimization than women who are in good health.

In Canada, using Statistics Canada's cycle 13 of the General Social Survey (1999), Brownridge concluded that women with disabilities had 40% greater odds of violence in relation to women without disabilities. Also, the result of this research showed that perpetrator-related characteristics alone accounted for the elevated risk of IPV (Brownridge, 2006). Recently, Tutty et al. (2021) examined in a triprovincial study how mental issues associated with IPV relate to women's intersecting identities of race/ethnicity, disability status, and child abuse history. They concluded that disability was only associated with more mental symptoms.

In South Korea, Kim and Lee (2016) examined the National Survey on Persons with Disabilities from 2011 and found that the risk factors for women living with disabilities were age, educational level, experience with discrimination, awareness of disability discrimination, external support, and satisfaction with the number of friends. Employment was not significant in this survey, maybe because 74.7% of respondents in the sample were largely unemployed.

To summarize, there are three aspects concerning IPV among women living with a disability and those living without a disability that the literature highlights. First, IPV-related violence is experienced only by women living with a disability. Second, that IPV may be experienced differently by women living with a disability and women living without a disability. And third, those women living with a disability experience IPV more frequently than women living without a disability. The frequency of violence that women living with disabilities suffered in comparison with women living without disabilities can be studied using a quantitative multilevel intersectional analysis, which we will explain in the next section.

## Multilevel intersectional analysis

Although IPV is one of the main problems in our society, we have just highlighted that “women are not a homogeneous group” (Balderston, 2013, p. 21). The concept of intersectionality refers to the relationship between complex social positions that affect identities and inequalities. Specifically, the authors refer to the relationship between inequalities of gender, disability, race, and other categories in individuals, social practices, institutional agreements, and cultural ideologies, together with the results of these interactions in terms of power (Crenshaw, 1989; McCall, 2005; Yuval-Davis, 2006; Davis, 2008; Cole, 2009; Anthias, 2013; Rodriguez Martínez, 2015; Brown, 2017; Hunt, 2018; Naples et al., 2019).

Since Crenshaw's analysis, there have been advances in the formulation of the intersectional approach, especially concerning the analysis of different levels (McCall, 2005; Choo and Ferree, 2010; Winker and Degele, 2011; Rodriguez Martínez, 2015). There are three levels in this formulation of the intersectional analysis: the anti-categorical, the intra-categorical, and the inter-categorical. In McCall's words, the first level of analysis refers to the anti-category complexity, which “is based on a methodology that deconstructs analytic categories” (2005, p. 1,773). The second level, the intra-categorical, “focuses on the intensive study of individual groups or cases” (2005, p. 1,782). In the first case, we can study how women living with a disability experience IPV and what they understand about IPV by comparing it with women without disabilities. It could be the kind of analysis that Baird et al. (2019) call intersectional lens; or the one that Rodriguez Martinez suggested to analyze IPV among women working in prostitution (Rodriguez Martínez, 2015; Rodriguez Martinez and Cuenca Piqueras, 2019). In that way, we can find the specific violence that women living with a disability suffered and which remains invisible when we compare it with the hegemonic model of women without disabilities. In the second case, we can study how the personal trajectories of women, living with a disability and experiencing IPV, develop.

The current quantitative intersectional analysis focuses on the third level of analysis. At the third level, inter-categorical, the intersectional approach tries to “document the relationships of inequality across social groups” (2005, p. 1,773). According to Winker and Dalege's work on this level, “strategic use is made of the categories and the relationships of multiple inequalities between socially constructed groups are analyzed” (Winker and Degele, 2011, p. 52–53). As summarized by Anthias, the inter-categorical level delves into the connections between categories (e.g., comparing data on gender and the ethnic composition of labor markets) (Anthias, 2013, p. 126).

In this research, we aimed to explore the specific combination of inequalities that affect European women living with a disability and compare them with women who live without a disability. Thus, the following questions are asked: Could it be said that the experience of IPV is something that can simply be added to the rest of the social categories or does this category interact with other social divisions? What is the result of these interactions?

From the intersectional approach, it is considered that in our societies, there is not an addition of the oppressions, but that there are certain social categories that can overlap with others. Is this the case of disability? In what way can we say that disability overlaps with other social divisions? We propose to control the combination of factors that increase the probability of suffering physical IPV in the general population of women, and see what social categories interact with living with a disability. To do that, we need an explanatory ecological framework of IPV to choose the variables we will incorporate in our analysis.

## Explanatory framework and variables

To select the variables to be included in our analysis, we suggest starting from the model proposed by Brownridge for studying intimate partner and ex-partner physical violence among women with disabilities (2006). The model recognizes an array of potential social divisions and organizes them within an ecological context into a framework based on whether they are related to the women, to the perpetrator, or to the relationship. The ecological model allows us to organize the characteristics of the women who suffered IPV and to broaden our view to include the social environment that surrounds them, which seems appropriate for our quantitative intersectional analysis.

Hence, we will work with three groups of variables to discover the risk factors that affect IPV among women living with and without a disability. The first factor refers to the women who suffered IPV. Here, we will include age (the older the women, the more likely they are to experience violence), educational level (the lower the educational level, the more likely it is to experience violence), socioeconomic status (the lower the status, the more likely it is to experience violence), and marital status (those who are separated will be more likely to experience IPV). The variables related to the characteristics of the perpetrator that we will use are as follows: alcohol consumption (the greater the level of consumption, the more violence there is) and partner's physical violence against others (the more violent he is toward other people, the more likely he is to be violent toward his partner). The third factor refers to the relationship of dependency in the couple. The variables we will include are the duration of the relationship (the shorter the relationship, the greater possibilities for physical violence), salary comparison (less than partner will increase the likelihood of IPV), and equal say concerning the use of household income (not having equal say increases the risk of IPV) (Brownridge, 2006; Zlotnick et al., 2006; Kai et al., 2018; Rodriguez Martinez and Cuenca Piqueras, 2019).

Other researchers have added that the violence suffered by women usually comes with more violence, so women who experience physical violence from their intimate partners probably have experienced other forms of violence from people who are not their intimate partners or ex-partners or when they were a child (Jones et al., 2012; Widom et al., 2014). Now that we have the explanatory framework and variables, we will describe the method of our study.

### The data set

Our analysis was carried out with the data from the European Survey on Violence against Women, by the European Union Agency for Fundamental Rights (FRA) between 2010 and 2012. In that survey, 42,002 women were interviewed, with a representative sample of women from each of the 28 countries of the European Union. In each country, a minimum of 1,500 women, aged between 18 and 74 and residents of the European Union, were interviewed. All the women interviewed were randomly selected, so that the results of the survey would be representative of the European Union as a whole and each of the countries individually. Face-to-face interviews were conducted with the women in their own homes using a standardized questionnaire prepared by the FRA from consolidated survey instruments. The interviews were conducted between April and September 2012.

Of the 42,002 women interviewed, a total of 2,100 answered that they considered themselves to be disabled (they answered yes to the question: “Do you consider yourself to be disabled?” while 39,902 declared that they did not consider themselves to be disabled. The survey did not ask about the type of disability the women suffered from or why they considered themselves to be disabled. The circumstance that it is the women who consider themselves disabled allows us to work with a definition of disability made by the women themselves, which meets one of the requirements suggested by feminist standpoint methods (Balderston, 2013). Considering oneself disabled does not imply that there is a social recognition of this disability. In fact, in a question about their employment situation, they are offered the answer that they are not working because of a disability. Of the women who responded affirmatively (*N* = 654), only 378 considered themselves to be disabled. As we noted before, living with a disability is a fluid social position.

In this survey, the percentage of women who reported having experienced some form of physical IPV (the answer yes to “My partner or an ex-partner has been physically violent against me”) was significantly higher among the group that considered themselves to be disabled. Of the women that consider themselves living with a disability, 24% reported that their partner or ex-partner had been physically violent against them. In the European Union, the percentage of women that suffered physical IPV and considered themselves not to be living with a disability was 15.1%.

Since the European survey was designed to obtain information on IPV experienced by women, there are sufficient variables that allow us to analyze the association with the prevalence of physical violence by an intimate partner or ex-partner in women who consider themselves to be disabled.

## Data analysis

To explore the physical IPV among women living with disabilities and to investigate the intersections with different social categories, the first stage consisted of descriptive analyses in which bivariate relationships were examined using cross-tabulations with chi-square significance tests. In the second stage, we carried out more elaborate analyses using logistic regression. Multiple logistic regression is an appropriate technique because we intend to predict a dichotomous dependent variable (My partner or an ex-partner has been physically violent against me) from a set of independent variables. This technique also has a relatively simple interpretation. For any given variable, it simply provides a ratio of the odds of IPV. The variable is positively related to IPV when the value of the odds is >1. The variable is negatively related to IPV if it is <1.

An important characteristic of modeling variables in logistic regression is that it allows us to measure the extent to which the estimate of the log odds of independent variable changes when controlling it for other independent variables. This allows us to observe how social categories interact. When the odds ratio of one variable is not constant over the level of another variable, the two variables are said to have a statistical interaction or effect modification. As Hosmer, Stanley, and Rodney point out, effect modification is used “to describe the fact that the log odds of one variable are modified or changed by the values of the other variable” Hosmer et al. (2013, p. 65).

We have built several models to explain physical IPV. In the first model, we have only introduced the “I consider myself to be disabled” variable and we have been adding the variables that have to do with the factors related to the women who suffered IPV, the aggressor, and the relationship. Finally, we introduced the variables related to the experience of physical violence before the relationship. At each of the levels, we manually checked the variables whose interaction was significant and added them.

## Results

### Socio-demographic of women living with/without a disability

Table 1 (Annexes) shows the percentages of women living with (or without) a disability according to their characteristics, the characteristics of their partner or ex-partner, those related to their relationship with their partner or ex-partner, and those related to their experience of IPV by someone other than their partner or ex-partner before and after the age of 15 years. In this table, we have only included the variables that show a significant difference between women living with a disability and those living without a disability, after performing a chi-square analysis. Race/ethnicity is not significant here.

**Table 1:** Descriptive of living with (without) a disability respondent.

**Variables**	**% Living without a disability (*N* = 39,902)**	**% Living with a disability (*N* = 2.100)**
**Partner or ex-partner physically violent against me**		
(Self-completion questionnaire: My partner or an ex-partner has been physically violent against me)		
Yes	15.1	24.0
**Finding with household income**		
(Which comes closest to how you feel about your household's income nowadays?)		
0. Finding it difficult or very difficult on present income	37.1	59.3
1. Living comfortably or coping with present income	62.9	40.7
**Educational Level**		
(What is the highest level of education you have achieved)		
0. Primary	27.6	45.9
1. Secondary	50.2	43.2
3. Tertiary	22.2	11.0
**Age**		
0. No more than 30.	17.2	2.8
1.More than 30	82.8	97.2
**Marital Status**		
(Are you currently married or in a civil partnership?)		
1. Married or in a civil partnership	60.9	51.2
2. No Married or in a civil partnership	39.1	48.8
**Partner drunk**		
(How often does your partner drink so much that he/she gets drunk?)		
1. No drunk usually	96.4	94.9
2. Drunk more than once a week	3.6	5.1
**Partner violent against other**		
(Has your partner ever been physically violent toward anyone outside the family?)		
0. no	94.8	94.2
1. yes	5.2	5.8
**Duration of the relationship**		
(Duration of the relationship)		
0. More than 10 years	67.5	83.2
1. Under 10 years	32.5	16.8
**Comparison with a partner earning**		
(Does your partner earn less than you, or are your earnings roughly the same, or does your partner earn more than you?)		
0. Same or more	87.6	89.4
1. Partner less	12.4	10.6
**Equal say in economic decisions**		
(Do you feel you have an equal say about the use of the household income?)	40.3	54.1
Yes	59.7	45.9
No	40.3	54.1
**Since 15 years, someone other physically violent against me**		
(Self-completion questionnaire: Since 15 years, somebody other than partner/ex-partner has been physically violent against me)		
0.No	89.8	84.1
1.Yes	10.2	15.9
**Rec When under 15 years old, somebody was physically violent against me**		
(L05. Self-completion questionnaire: When under 15 years old, somebody was physically violent against me)		
0. No	87.2	79.7
1. Yes	12.8	20.3

Women who live with a disability have experienced IPV by their partner at 15.1%, 9.1 percentage points higher than those who do not consider themselves to be living with a disability. Compared to women living without a disability, those living with a disability are characterized by lower income, lower educational level, and older age. Compared with women living without a disability, it is significantly more common not to be married or to be living in a relationship. If we look at the variables related to the partner or ex-partner, women living with a disability state that their partners abuse alcohol and are physically violent to anyone outside the family more often. In terms of the relationship-related variables, women living with a disability are in much longer relationships, with men who earn the same or more than they do, and in which they feel that they do not have equal say concerning the use of household income. The last two variables show that women living with a disability have experienced physical violence by other people who were not their partner or ex-partner more frequently than women living without a disability, both up to the age of 15 years and after the age of 15 years.

This first description suggests that women with disabilities may experience other forms of violence that we cannot easily capture with quantitative studies, or even that the way they experience IPV is different from the women who live without a disability. With quantitative data, we can work with the disability category to see how it combines with other social divisions in terms of the characteristics of the women who suffered IPV, those of the aggressors, the relationship, and their previous history of physical violence. To do so, we will analyze the full sample and see what the odds of suffering violence are when living with a disability.

### Odds ratios associated with IPV

Table 2 shows the odds ratios of the IPV logistic regression only taking disability as the independent variable (model 1), to which we have added the variables related to the women who suffered IPV (model 2) in addition to its interactions (model 3), the variables related to the aggressors (model 4) and its interactions (model 5), the variables related to the relationship (model 6) and its interactions (model 7), and the history of physical violence before the relationship (model 8) and its interactions (model 9). All the models were statistically significant at *p* < 0.001. The Pseudo R2 of the models goes from 0.004 in the first model to 0.245 in the ninth model, which means that the last model explains 24.5% of the variation of physical IPV.

**Table 2:** Odds ratios from logistic regression on “My partner or an ex-partner has been physically violent against me” (*N* = 42,002).

	**Model 1 (*R*^2^=0.004)**	**Model 2** **(*R*^2^ =0.021)**	**Model 3** **(*R*^2^ = 051)**	**Model 4** **(*R*^2^ =0. 090)**	**Model 5** **(*R*^2^=0.091)**	**Model 6** **(*R*^2^ =0.115)**	**Model 7** **(*R*^2^ = 0. 116)**	**Model 8** **(*R*^2^ = 0.244)**	**Model 9** **(*R*^2^ = 0.245)**
**Disability: no is reference, yes**	1.769^***^	1.367^***^	1.704^***^	1.503^***^	1.496^***^	1.584^***^	1.589^***^	1.300^**^	1.343^**^
Finding with household income: comfortable is reference, difficult		1.543^***^	1.580^***^	1.538^***^	1.447^***^	1.379^***^	1.311^***^	1.303^***^	1.350^***^
Educational level (tertiary is the reference), Secondary		1.107^*^	1.105^***^	1.256^***^	1.257^***^	1.445^***^	1.449^***^	1.538^***^	1.536^***^
Primary		1.191^***^	1.188^***^	1.215^***^	1.217^***^	1.307^***^	1.310^***^	1.359^***^	1.360^***^
Age (less than 30 reference), more than 30		1.838^***^	1.839^***^	2.073^***^	2.076^***^	2.818^***^	2.802^***^	2.704^***^	2.714^***^
Marital status. Married is reference, no married		2.100^***^	2.103^***^	2.693^***^	2.702^***^	1.480^***^	1.484^***^	1.414^***^	1.420^***^
**Finding difficulty with household income by Disability**			1.422^***^						
Partner drunk: Never drunk is reference, drunk				1.823^***^	1.818^***^	1.761^***^	1.759^***^	1.638^***^	1.622^***^
Partner Physically violent against other (no is the reference), yes				2.710^***^	2.222^***^	2.231^***^	2.242^***^	1.787^***^	1.776^***^
**Finding difficulty with household income by Partner Physically violent against other**					1.609^***^	1.413^**^	1.391^**^	1.395^**^	1.405^**^
Duration of the relationship: more than 10 years is reference, under 10 years						2.219^**^	2.217^***^	2.137^***^	2.143^***^
Comparison with a partner earning: some o more is reference; less than the partner						1.199^***^	1.203^***^	1.160^**^	1.157^**^
Equal say (yes is reference), no						1.702^***^	1.558^***^	1.476^***^	1.567^***^
**Equal say by Finding difficult with income**							1.225^**^	1.169^*^	
Since 15 years: somebody physically violent against me: (no is the reference), yes								5.621^***^	5.616^***^
Under 15 years: somebody physically violent against me: (no is the reference), yes								2.480^***^	2.552^***^
**Under 15 years: somebody physically violent against me by disability**									1.572^**^
**Partner Drunk by disability**									3.144^***^

*** P < 0.001,

** p < 0.01,

* p < 0.1.

Nagelkerke R of model.

Model 1 shows that among European women, who consider themselves living with a disability, the odds of suffering physical violence by a partner or ex-partner is increased by 1.769. In model 2, we have included the socio-demographic variables referring to the women who suffered IPV (finding difficulty with household income, educational level, age, and marital status). Controlling for the rest of the variables included in the equation, the odds of living with a disability drop to 1.367. In this equation, finding it difficult to live on current income increases the odds of suffering violence by 1.543; the levels of secondary and, above all, primary educational level also increase the odds of suffering IPV by 1.107 and 1.191; being over 30 years old increases the odds of suffering IPV by 1.838; not being married or living with one's partner increases the odds of suffering violence by 2.100. Thus, it looks like marital status, age, and income increase the odds of suffering IPV more than disability in this model. Nevertheless, its interactions were missing.

As we mentioned earlier, we manually entered all possible interactions between the variables, but the only interactions that were significant were finding it difficult to live on present income and living with a disability. Model 3 includes that interaction and, as it can be seen in the table, improves our predictive ability. In this case, and controlling for the rest of the variables, living with a disability increases its odds to 1.704 and the interaction between income and disability increases by 1.422. Finding difficulty with household income increases its odds also slightly (1.580) and the rest of the variables remain more or less with the same odds. These results suggest that living with a disability should be considered as a category to be analyzed separately, and also how it interacts with income. In other words, finding economic adversity interacting with a disability generates a plus of suffering IPV that allows us to improve the overall predictive model when analyzing the individual factors of physical IPV. Living with a disability and finding it difficult to live on present income are intersectional social divisions in the production of physical IPV when we analyze the socio-demographic characteristics of the women who suffered IPV. This interaction will not be significant when we include the variables related to the partner or ex-partner.

We have introduced two variables related to the partner or ex-partner in model 4 (partner who abuses alcohol and partner who is physically violent against others). Controlling for the rest of the variables, partner who abuses alcohol has an odd of 1.823 and partner who is physically violent against others has an odd of 2.710. As a consequence of this introduction, the odd of “not married” (2.693), as well as the odd of “over 30 years old” (2.073) and the odd of educational level, especially secondary (1.256), also increase. However, the odds of living with a disability and difficulty of income decrease, when we control with aggressors' characteristics.

In model 5, we have added the interaction between difficulty of income and a violent partner or ex-partner toward others. As a consequence of this interaction, there are small variations in the odds of the other variables. We can say that a violent partner against others increases the odds of suffering violence by 2.222, and by 1.609 more because income interacts with a violent partner toward others.

In model 6, we added the relationship-related variables. Relationships under 10 years double the odds of suffering physical IPV and earning less than the partner increases the odds slightly (1.199). But the relationship-related variable whose odd is highest is the one referring to unequal say concerning the use of income in the household, which reaches 1.702. In this model, which allows us to control for the characteristics of the women who suffered IPV, the aggressor, and the relationship, the odds of living with a disability increase slightly from the previous model.

Unequal say about income also interacts with income, as it can be seen in model 7, where it reaches an odd of 1.225. In other words, there is an additional possibility of suffering physical IPV when there is a lack of equality in the relationship to decide on income, but the interaction between low income and lack of equality in decisions also adds another additional odd of suffering physical IPV. For the rest of the variables, we can observe that when controlling for those that refer to the relationship, educational level, and age, their odds increase considerably, and marital status decreases.

The last two models show a complete picture, as we have added the variables related to the experience of physical violence by people other than the partner or ex-partner, “under” and “over the age of 15.” These two variables have very high odds. The odds of suffering violence when the woman has experienced physical IPV from the age of 15 years is 5.621, and that before the age of 15 years is 2.480. In the final model, we introduce two more interactions: those between having suffered violence before being 15 years of age and considering oneself disabled, which has an odd of 1.572. Also, a partner who abuses alcohol and is disabled has an odd of 3.116.

## Discussion

As expected, living with a disability, low income, low educational level, advanced age, and not being married increases the probability of suffering physical IPV. In addition, a partner who abuses alcohol and is violent toward others also increases the probability of physical IPV. As for the relationship, short relationships, where the woman earns less than the partner and where there is no equality in decision-making, increase the likelihood of physical IPV. The final model shows that the factor that most triggers the odds of suffering physical IPV is if there is a previous experience of physical violence by other people, especially if it occurred after the age of 15 years.

But interactions must be added. Living with a disability interacts with low income.

Low income also interacts with the partner who triggers violence toward others, but also significantly increases inequality in decision-making about what to do in the household. In addition, partners being violent toward others triggers the likelihood of IPV, especially when it occurs in a low-income setting. There is also another plus in the interaction of disability with the experience of violence when the woman was under 15 years, and between living with a disability and the partner getting drunk.

As the literature highlights, women living with a disability experience more IPV than women living without a disability, and this violence can be different and specific. In our study, 24% of women living with a disability suffered physical IPV. That percentage is only one point higher than that detected in the USA (Harrell, 2015).

In the first approach of intersectional analysis, Crenshaw argued that the inequalities that black women experienced were invisible since they did not coincide with those of their black partners or with those of white women (Crenshaw, 1989). In the case of women living with a disability who experience IPV, the type of violence would be what Austin et al. (2014) call “disability-specific abuse.” Our research focused on the third level of analysis.

Living with a disability almost doubles the odds of IPV. But it also interacts particularly with variables related to income, with the partner being violent toward other people, and abusing alcohol. It is important to highlight this point because studies on IPV indicate that disability is a risk factor among women, as is low income. But it is not often emphasized that the two together create a new odd of suffering IPV that needs to be taken into account. In terms of social policies, this result should induce investment in IPV prevention policies among poor women who live with a disability, who have a history of physical violence, and whose partners abuse alcohol. Those are the women who are at a bigger risk of abuse and who could maybe inhibit their capacity to respond.

The ecological model proposed by Brownridge (2006) allowed us to organize the variables related to IPV on the victim-related characteristics, the perpetrator-related characteristics, and the relationship factors. To these factors, we have added the previous experience of physical violence against women. Our analysis corroborates Brownridge's results, since the variables we have introduced referring to the characteristics of the perpetrators (in our case, drunk and violent against others) significantly improve our model. But, in addition, we have shown that, as far as perpetrators are concerned, there is also an interaction between violence against others and income. That is, violent perpetrators against others living in low-income households add to the likelihood of IPV. Moreover, as we highlighted earlier, the variables in our analysis that mostly triggered the odds of suffering violence are those related to the previous experience of the women who suffered IPV, so we corroborate the results of Widom et al. (2014).

Our final model has reduced the odds of living with a disability for IPV from 1.769 to 1.343 after controlling for the rest of the variables. That reduction supports the authors who pointed out that disability is about a person's relationship to other social divisions in the social environment as a whole (Garland-Thomson, 2005; Shakespeare, 2014; Muster, 2020) or “the intersectional nature of violence” (Balderston, 2013). But we have not been able to reach a model in which living with a disability does not increase the odds of suffering IPV controlling for the rest of the variables. That is, disability is not just an intersectional category. It is also a source of inequality and oppression regardless of other social divisions.

## Limitations

Our analysis has many limitations. With this survey data, we cannot work on the anti-categorical and inter-categorical levels of analysis. These two levels of analysis cannot be explored with quantitative techniques, among other reasons, because the IPV surveys do not ask women about their sex-gender identifications nor about the effects of moving from living without a disability to living with a disability, and the effect that it has in terms of personal experiences of suffering IPV.

Another limitation has to do with the definition of “living with a disability,” The survey we are handling does not ask any question that permits us to differentiate between physical or psychiatric disability. We may be talking mainly about women who experience a physical disability. We also do not know why they say they feel part of the group of people who live with a disability.

Nor can we determine the causal relationship between living with a disability, economic vulnerability, experience with other violence, and experiencing IPV. For example, it is possible that living with a disability leads to experiencing greater economic vulnerability, or that economic vulnerability leads to living with a disability, and so on. It is possible that living with a disability leads to experiencing more IPV. But also that physical IPV leads to living with a disability. Qualitative research could help to answer these questions.

## Data availability statement

The original contributions presented in the study are included in the article/supplementary material, further inquiries can be directed to the corresponding authors.

## Author contributions

PR is the author of this article. It means that she has contributed to the conception, design of the study, and did the analysis of the database, performed the statistical analysis, and drafted the manuscript. She also did the manuscript revision, read, and approved the submitted version.

## Conflict of interest

The author declares that the research was conducted in the absence of any commercial or financial relationships that could be construed as a potential conflict of interest.

## Publisher's note

All claims expressed in this article are solely those of the authors and do not necessarily represent those of their affiliated organizations, or those of the publisher, the editors and the reviewers. Any product that may be evaluated in this article, or claim that may be made by its manufacturer, is not guaranteed or endorsed by the publisher.
